# Modernising the MSE: Embedding Eating and Nutritional Assessment Into the 21st-Century Mental State Examination

**DOI:** 10.1192/bjo.2026.11343

**Published:** 2026-06-30

**Authors:** Edwin Birch, Agnes Ayton, James Downs

**Affiliations:** 1 Oxford Health NHS Foundation Trust, Oxford, United Kingdom; 2 Independent, United Kingdom

## Abstract

**Aims:**

To review the utility of the traditional Mental State Examination (MSE) in identifying feeding or eating disorders (FEDs), and to propose a modernised approach that incorporates assessment of eating behaviours, nutrition, weight, and body image disturbance to improve detection and patient safety.

**Methods:**

A narrative review of existing MSE frameworks was undertaken, examining their historical development and relevance to contemporary psychiatric presentations. The unique diagnostic challenges of FEDs were considered, alongside common barriers to detection such as stigma, ego-syntonic psychopathology and structural inequalities in the medical establishment. Our co-author provided lived experience accounts to provide a patient-led perspective on the limitations of the MSE in modern practice. Based on these gaps, a proposal was developed to integrate semi-structured questions on diet, weight, and compensatory behaviours into routine MSE practice.

**Results:**

The emphasis of the current MSE is seen to be heavily weighted towards traditional psychiatric presentations such as psychosis and affective disorders, inadequately capturing key psychopathology associated with FEDs such as eating behaviours, nutritional status, andbody image disturbance. This omission risks delayed diagnosis and compromised patient safety. Incorporating targeted, semi-structured questions into the MSE offers a feasible and systematic way to improve early recognition of FEDs across diverse populations. Additional benefits will be gained in safe psychotropic drug monitoring with early recognition of nutritional side effects.

**Conclusion:**

Modernising the MSE to explicitly include assessment of eating behaviours and related psychopathology may enhance early detection of FEDs, reduce stigma through routine enquiry, and improve clinical outcomes and patient safety. Updates to medical training and everyday psychiatric practice are required in tandem to reflect the diverse presentations of modern mental health disorders.

